# Assessment of asymmetric leg loading before and after total hip arthroplasty using instrumented shoes

**DOI:** 10.1186/1743-0003-11-20

**Published:** 2014-02-28

**Authors:** Alicia Martínez-Ramírez, Dirk Weenk, Pablo Lecumberri, Nico Verdonschot, Dean Pakvis, Peter H Veltink

**Affiliations:** 1 Mathematics Department, Public University of Navarra, Pamplona, Spain; 2 Institute for Biomedical Technology and Technical Medicine (MIRA), University of Twente, P.O. Box 217, Enschede 7500 AE, The Netherlands; 3 Orthopedic Research Laboratory, Radboud University, Nijmegen Medical Centre, Postbox 9101, Nijmegen 6500 HB, Netherlands; 4 Department of Orthopedic Surgery, Medisch Spectrum Twente, PO Box 50000, Enschede 7500 KA, Netherlands

**Keywords:** Instrumented shoes, Gait parameters, Sit to stand parameters, Total hip replacement, Ground reaction forces

## Abstract

**Background:**

Total hip arthroplasty is a successful surgical treatment in patients with osteoarthritis of the hip. Different questionnaires are used by the clinicians to assess functional capacity and the patient's pain, despite these questionnaires are known to be subjective. Furthermore, many studies agree that kinematic and kinetic parameters are crucial to evaluate and to provide useful information about the patient’s evolution for clinicians and rehabilitation specialists. However, these quantities can currently only be obtained in a fully equipped gait laboratory. Instrumented shoes can quantify gait velocity, kinetic, kinematic and symmetry parameters. The aim of this study was to investigate whether the instrumented shoes is a sufficiently sensitive instrument to show differences in mobility performance before and after total hip arthroplasty.

**Methods:**

In this study, patients undergoing total hip arthroplasty were measured before and 6–8 months after total hip arthroplasty. Both measurement sessions include 2 functional mobility tasks while the subject was wearing instrumented shoes. Before each measurement the Harris Hip Score and the Traditional Western Ontario and McMaster Universities osteoarthritis index were administered as well.

**Results:**

The stance time and the average vertical ground reaction force measured with the instrumented shoes during walking, and their symmetry index, showed significant differences before and after total hip arthroplasty. However, the data obtained with the sit to stand test did not reveal this improvement after surgery.

**Conclusions:**

Our results show that inter-limb asymmetry during a walking activity can be evaluated with the instrumented shoes before and after total hip arthroplasty in an outpatient clinical setting.

## Background

Total hip arthroplasty (THA) is one of the most effective surgical procedures in orthopedics to relieve hip osteoarthritis (OA) that results in a significant improvement in functional capacity of patients [1]. OA is the clinical and pathological outcome of a range of disorders characterized by structural, and eventually symptomatic, failure of one or more synovial joints [2].

OA of the hip is a common and frequent disease. Ten percent of the population older than 60 years have important clinical problems attributed to osteoarthritis [3]. The advanced stage of OA is characterized by severe pain as the predominant symptom [4,5]. Individuals will have limitations that impair their ability to perform activities of daily living (ADLs) [6].

After THA, patients usually perceive a dramatic pain relief. However, their motor skills do not reach the normal level [7]. During the rehabilitation program, the goal is to minimize postoperative complications and to maximize the functional status of the patient. In addition, it is important to evaluate pain, mobility performance, activities of daily living, and overall satisfaction and welfare of the patient. Clinicians use validated questionnaires to assess and compare the patient’s condition before and after THA [8-11]. The Harris Hip Score (HHS) and the Traditional Western Ontario and McMaster Universities osteoarthritis index (WOMAC) are the most used and relevant questionnaires [12,13]. Gait velocity has been measured in several studies as a method to assess the functional capacity in patients with OA [12,14]. It has been reported that walking speed increases significantly during the post-operative rehabilitation [12]. The questionnaires are not based on objective physical measurements and depend on the subjective opinion of both the patient and clinician. In addition, the questionnaires and the gait velocity do not provide information about the movement patterns underlying the functional capacity. Consequently, there is a clinical necessity for objective physical measurements to evaluate the functional progress after THA.

Walking and sit to stand (STS) movements are basic motor activities relevant to evaluate the functional effects before and after THA [14,15]. They are, therefore, evaluated and included in the HHS [8] and WOMAC [12].

Gait analysis is a useful tool for assessing functional deficits in patients before and after THA [16-18]. During walking, weight loading asymmetry can be quantified measuring the left-to-right difference in vertical ground reaction force [7,19] to identify atypical limb loading for individuals before and after THA [19-21]. Many studies agree that it is crucial to clarify the factors influencing the improved walking after THA to provide useful information about the patient’s evolution for clinicians and rehabilitation specialists [12].

The STS movement has been accepted as a prerequisite for successful gait performance [22] and it is considered an important and demanding task in our daily life [23,24]. Its performance involves large movement amplitudes in hip muscles to produce sufficient power to lift the body mass [25]. Amongst people with hip OA, asymmetric limb loading seems to be present while they perform STS movements, with significant differences between patients and controls [7,14], who perform the task with a comparable contribution from each lower limb [26].

Currently, objective functional mobility assessment can only be performed in a specialized laboratory, using force platforms and optical systems [17,18,27]. These laboratory measurement systems are expensive and not generally available in orthopedic practice. Moreover, the area of the force platform restricts the range of motion and the number of consecutive steps that can be measured. Optical systems also show restrictions since the line of sight can be easily blocked [28]. More portable and low-cost methods to quantify functional aspects of patients are dynamic Emed and pedar systems which can be used to measure plantar pressure during static and dynamic activities [29,30]. Furthermore, gait mats can also be used to provide spatial and temporal gait parameters [29,30]. These systems have the limitation that they measure only a small number of consecutive strides, and have a limited temporal and spatial resolution and are unsuitable to measure ground reaction forces and gait patterns.

A new ambulatory movement analysis system for kinetic and kinematic measurements should open new perspectives. Instrumented shoes (IS) are suitable for the measurement of ground reaction forces, position and orientation of the foot during walking and other tasks [28,31-33]. In this study, we explore the potential applicability of joint ground reaction force and inertial movement sensing by instrumented shoes to evaluate the functional progress after THA.

Our aim was to investigate whether instrumented shoes, sensing ground-reaction force and foot kinematics, are a sufficiently sensitive instrument to show differences in mobility performance before and after THA in an outpatient setting.

## Methods

### Subjects

Nineteen patients with hip OA participated in this study (eleven females and eight males, age: 62 (mean) ± 9 (SD) years, body mass 84.9 ± 10.8 kg and height 1.71 ± 0.08 m).

Patients were recruited from Medisch Spectrum Twente (Enschede, the Netherlands). These patients were scheduled to receive a primary THA. They were measured twice: before and 6–8 months after the surgery.

The inclusion criteria were primary unilateral osteoarthritis of the hip and a THA planned within the next 4 months and age between 50 and 80 years.

The exclusion criteria were any kind of leg arthroplasties, rheumatoid arthritis, a contra-lateral THA, any neurological disorder, other degenerative diseases or the inability to understand instructions or the questionnaires.

The study protocol was approved by the Medical Ethics Committee (METC) of the Medisch Spectrum Twente, (Enschede, the Netherlands) and full written consent was obtained from all participants.

### Data collection procedures

The measurement sessions were performed in the department of Orthopedic Surgery at the Medisch Spectrum Twente.

Both measurements, before [34,35] and after THA, included 2 functional mobility tasks, walking and a Sit-to-Stand test, while the subject was wearing instrumented shoes.

### Walking

Subjects were instructed to walk repeatedly at their preferred speed through a corridor between a predefined start and end point, 10 m apart at a constant speed. The measurement protocol is described in a pre-surgery study with 22 patients with hip OA [34]. Three of these 22 patients could not participate in the current post-surgery study because of diverse health-related reasons. In order to control the initial relative positions of the feet, the subject was asked to position the feet against a line on the floor before each walking trial. Three successful trials were collected per subject. The subject had to start 2.5 meters before the start mark and walk 2.5 meters past the finish mark. A stopwatch was started as soon as the subject’s foot crossed the start line and the timing was stopped when the person’s second foot crossed the finish line. In this way, the average gait velocity for all trials was calculated independently from the instrumented shoes as distance walked divided by walking time (gait velocity (GV) = distance/time).

### Sit-to-stand

Subjects were seated in a chair with armrests as it is described in a pre-surgery study with this patient group [35]. The chair height and depth were adjusted in a way that the knee angles were 90 degrees in a seated position. The subjects’ ankles were placed vertically under the knee. The subjects were asked to look straight forward and to rise at their own preferred speed with their arms folded across the chest after the “1, 2, 3, and rise” command. The subjects were instructed to stand quietly in the anatomical position for 5 s after each trial [22]. The placement of the seat and the position of the feet were marked on the floor with surgical tape to guarantee the same starting position in every trial. It was tested whether the subjects were able to stand up without using the armrests before the trial. If the subject was not able to perform the trial without using the armrests, he/she was allowed to use his/her arms. Three successful trials were collected per subject.

### Questionnaires

Subjects were asked, with the researcher’s supervision, to complete 2 questionnaires that are validated to evaluate hip function in THA patients: the Dutch version of the HHS [8], and the WOMAC [9].

### Instruments used

The ambulatory measurement system used in this study consisted of a couple of instrumented shoes (Xsens Technologies B.V., Enschede, the Netherlands) for 3D measurement of forces and torques under the feet, as well as 3D kinematics of the feet. The measured data was sent via wireless to a PC or laptop (Xbus master).

**Figure 1:**
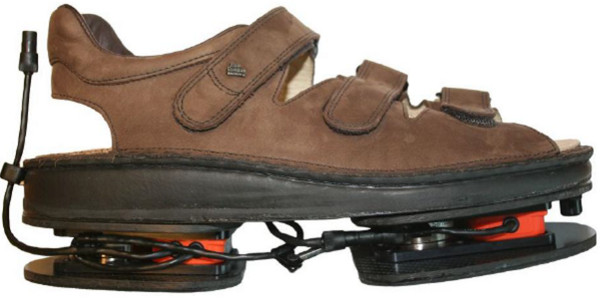
**Instrumented shoes.** Instrumented Shoes (right shoe). Each Force Shoe (left and right) has 2 sensors modules: one under the forefoot and one under the heel. A sensor module includes a Force/Torque Sensor and a Motion Tracker.

The instrumented shoes (Figure 1) are adjustable for shoe size. The signals were sampled at 50 Hz.

These instrumented shoes have been validated and successfully used before in different studies [28,36,37]. It has been demonstrated that they provide reliable and accurate measurements of 3D-ground reaction force, position and orientation [38]. Moreover, Van den Noort JC et al. have demonstrated that IS are suitable for the measurement of ground reaction forces in patient with OA [33,37]. The measurement system was calibrated before the measurement sessions using the method described by Faber et al. [39].

In the previous study, we found that the walking velocity decreased by 9% when patients walked with the instrumented shoes [34]. Consequently, as Van Den Noort et al. found [33], the influence of instrumented shoes characteristics on the gait pattern is small compared to normal intra-subject variability and the decrease on gait velocity due to wearing the instrumented shoes could be regarded as below clinical relevance.

### Data analysis

**Figure 2:**
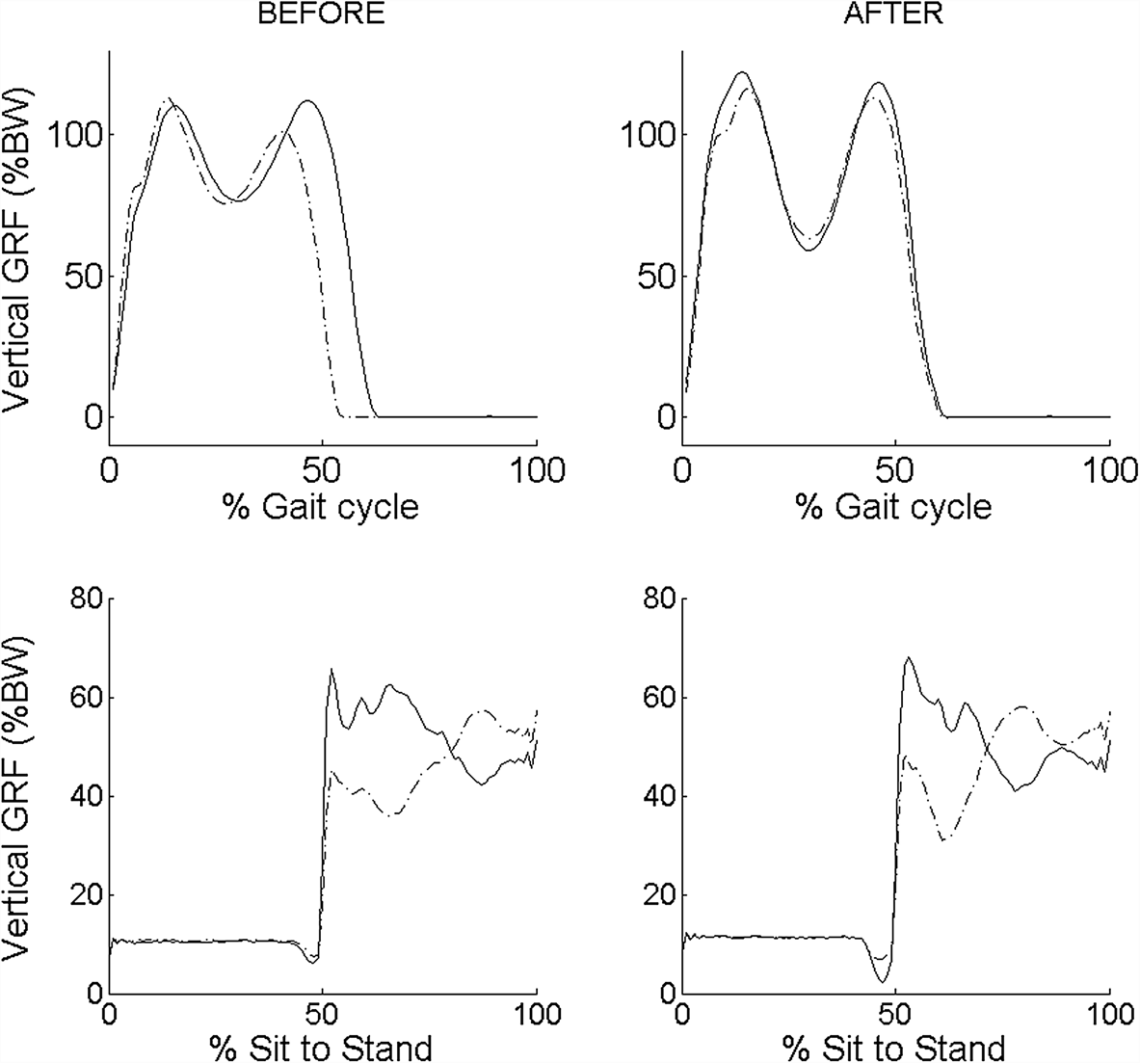
**IS parameters.** Vertical ground reaction forces during walking and sit to stand test: mean and standard deviation (SD) of the vertical ground reaction force of all trials, before (left part) and after (right part) THA for one representative subject during walking (up) and STS (down) test.

All IS parameters were further processed using MATLAB. The IS parameters were calculated for both involved and uninvolved legs. Ground reaction forces were normalized to body weight (BW) and reported as a percentage of body weight (%BW). Our analysis is restricted to the vertical ground reaction force signal, which is heavily influenced by the movement pattern. Vertical ground reaction forces during walking and sit to stand test before and after THA for one representative subject are plotted in Figure 2. Among all possible IS parameters, the following parameters were selected based on the previous studies of pre-surgery assessment with these patients [34,35].

### Walking

Time parameters include the stance time (t_stance_), defined as the % of cycle that the reference limb is in contact with the floor and the midstance time. In addition, the average vertical Ground Reaction Force (vGRF) for each involved and uninvolved leg was evaluated during the stance time (A_vGRF,w_) normalized by body weight (%BW).

### Sit to stand

The rise time t_rise_ is defined as t_rise_ = t_2_-t_1_, with, t_1_ being the time at which the sum of vGRF for both limbs first exceeds the initial vGRF level measured while the patient was sitting, and t_2_ being the time when the sum of vGRF for both limbs reaches body weight for the first time before attaining its maximum value.

Maximum peak of GRF (P_vGRF,sts_) was calculated for vGRF. In addition, the dynamic area (D_vGRF,rise_) defined as the area under the vGRF during rise time from t_1_ to t_2_, was included in the analysis.

**Symmetry index** (involved/uninvolved) (SI): The symmetry index was calculated using the Equation 1:

(1) $$\mathrm{SI}=\frac{\left(V_{I}‐V_{U}\right)}{V_{U}}*100\%$$

Where V_U_ and V_I_ are any of the aforementioned parameters for the uninvolved and involved leg respectively. Perfect symmetry results in SI = 0 (V_U_ = V_I_); positive and negative values indicate a greater asymmetry towards the involved and uninvolved limbs, respectively.

### Statistical analysis

The values for each parameter were averaged for all tests performed under the same condition, separating the involved limb of the OA patients from the non-involved limb.

Descriptive statistics of velocity and IS parameters, mean and standard deviation, were calculated. Paired sample t-tests were calculated to assess whether IS are sufficiently capable of indicating significant differences between before and after THA with a significance level of 0.05. In our case, since we have only 19 patients, the *Shapiro*-*Wilk test* was used to check the assumption of normality before the t-test.

## Results

Data from 19 patients (the average of the three trials per subject) were measured before and after THA. The assumption of normality was satisfied for all estimated parameters.

### Gait velocity and questionnaires outcomes

Mean, standard deviation and the statistic p-value for the comparison between before and after THA of gait velocity, Harris Hip Score and WOMAC outcomes are shown in Table 1.

**Table 1:** Gait velocity HHS and WOMAC outcomes measures in subjects before and after THA (N = 19)

		**Before THA**		**After THA**		
		**Mean**	**Sd**	**Mean**	**Sd**	**p_value**
**Gait velocity**		0,92	0,24	1,14	0,26	7,38E-07 Ɨ
**Harris hip score**		52,00	17,09	86,53	13,13	6,81E-08 Ɨ
**Womac**	Total	49,61	13,59	14,42	17,41	7,00E-08 Ɨ
	Pain	10,05	3,94	1,53	2,80	2,79E-08 Ɨ
	Stiffness	4,79	1,90	1,95	1,75	2,38E-05 Ɨ
	Physical functioning	34,77	9,16	10,99	14,04	4,95E-07 Ɨ

Ɨ Indicates significant differences between groups (p < 0.05).

### IS parameters

Mean, standard deviation and the statistic p-value for the comparison between before and after THA of instrumented shoes parameters are shown in Table 2.

**Table 2:** Mean ± standard deviation and p-value of instrumented shoes parameters during walking and STS tasks before and after THA (N = 19)

		**Involved**			**Uninvolved**		
		**Before**	**After**	**P-value**	**Before**	**After**	**P-value**
Walking							
	t_stance_	60.57 ± 4.04	61.10 ± 1.90	0.45	63.22 ± 3.27	61.15 ± 2.55	0.008
	A_vGRF,w_	73.15 ± 4.58	76.05 ± 2.71	0.0003	76.58 ± 3.84	77.88 ± 3.37	0.02
Sit to stand							
	P_vGRF,sts_	53.13 ± 6.85	55.76 ± 4.23	0.2234	64.29 ± 7.22	64.47 ± 6.71	0.9168
	D_vGRF,rise_	13.47 ± 21.94	13.31 ± 21.30	0.9296	18.66 ± 31.03	14.79 ± 22.20	0.2209

The patients showed significantly larger stance time before THA compared to after, for the uninvolved lower limb (p = 0.008) whereas they did not show any significant difference for the involved leg.

The patients showed significantly greater average vGRF during walking for both involved and uninvolved lower limbs after THA (p < 0.001 and p = 0.02 for the involved and uninvolved limbs, respectively).

There were no significant differences in IS parameters measured during the STS test before and after THA.

### Symmetry parameters

Mean, standard deviation and confidence intervals (C.I.) of the symmetry index of the IS parameters before and after THA are shown in Table 3.

**Table 3:** Mean, standard deviation (SD) and confidence invervals (C.I.) of the symmetry parameters during walking and STS tasks before and after THA

		**Before THA**		**After THA**	
		**Mean (SD)**	**C.I.**	**Mean (SD)**	**C.I.**
**Walking**					
	t_stance_	−4.03 ± 6.84	[−7.33,-0.73] Ɨ	0.02 ± 3.48	[−1.66,1.69]
	A_vGRF,w_	−4.47 ± 3.78	[−6.30,-2.64] Ɨ	−2.3 ± 2.25	[−3.38,-1.21] Ɨ
**Sit to stand**					
	P_vGRF,sts_	−15.81 ± 15.24	[−23.64,-7.97] Ɨ	−12.02 ± 12.39	[−18.39,-5.65] Ɨ
	D_vGRF,rise_	−26.67 ± 24.40	[−39.22,-14.12] Ɨ	−19.35 ± 21.33	[−30.31,-8.39] Ɨ

Ɨ Indicates significant differences between groups (p < 0.05).

The symmetry index of walking parameters, t_stance_ and A_vGRF,w_, reveals significant asymmetry before surgery (p = 0.02 and p < 0.001 respectively). After THA, t_stance_ did not show asymmetry (p ≤ 0.05) anymore. However, A_vGRF,w_ was still significantly asymmetric (p < 0.001) after surgery.

Symmetry index parameters of STS test, P_vGRF,sts_ and D_vGRF,rise_, were significantly asymmetric in both cases, before (p < 0.001 and p < 0.001, respectively) and after THA (p = 0.001 and p = 0.002, respectively).

**Figure 3:**
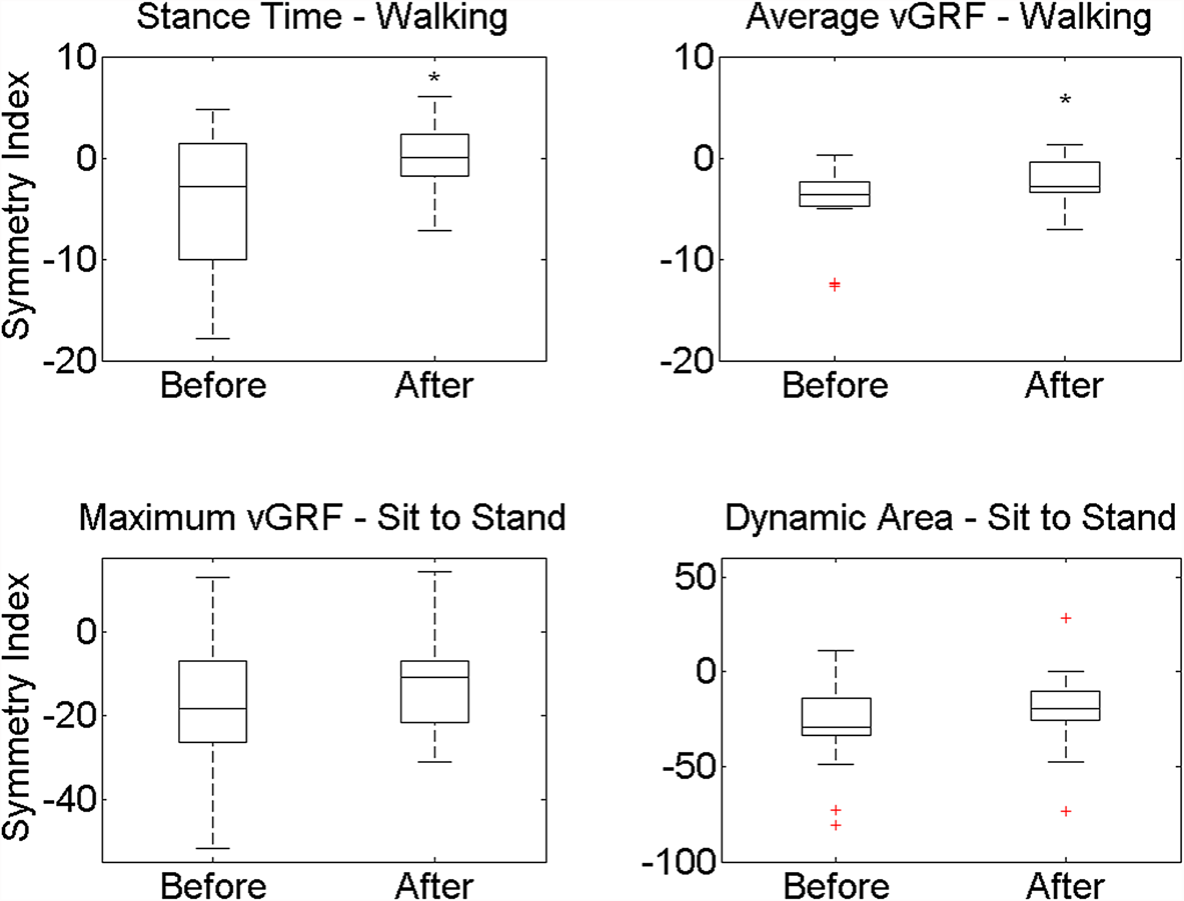
**Boxplot of SI during walking.** Boxplot of symmetry index of walking and sit to stand parameters for all patients. The box indicates the lower and upper quartiles with the central line showing the median. The top and bottom lines of the box represent, respectively, the medians for the upper and lower halves of the data and the ‘cat’s whiskers’ represent the highest and lowest values of the distribution, excluding outliers (+). *symbol represents significant differences between before/after THA.

Symmetry index of walking parameters, t_stance_ and A_vGRF,w,_ were significantly different before and after THA (p = 0.01 and p = 0.03, respectively). On the other hand, there were no significant differences between symmetry index of STS parameters before and after THA (p ≤ 0.05). Boxplots of the symmetry index of the IS parameters during walking and STS test before and after THA are plotted in Figure 3. The symmetry index of t_rise_ during STS test is not included in the figure because this parameter is computed from the sum of vGRF for both limbs, as mentioned in the methods section.

As can be seen in Figure 3, boxplots show a large variability of symmetry parameters influenced by inter-individual differences. These differences deviate considerably less from zero after THA.

## Discussion

This study shows that inter-limb asymmetry can be evaluated with instrumented shoes, which show enough sensitivity to reveal differences in mobility performance before and after total hip arthoplasty in an outpatient setting. Stance time, average vertical GRF and their symmetry indices measured with the instrumented shoes during walking showed significant improvement after THA. Reproducing the result of the meta-analysis of Vissers et al. [12], gait velocity and questionnaires outcomes also showed significant differences before and after THA. However, the instrumented shoes parameters measured during the STS test did not show significant improvement after surgery.

Harris Hip Score and WOMAC scores show that patient’s condition improves significantly during the 6 to 8 months after THA. This result is in agreement with those of Vissers and Lavernia [12,40] where it is reported that these questionnaires are a valid method to evaluate patient satisfaction and the quality of life achieved, showing significant improvement after THA. These questionnaires are easy to administer, simple to understand for patients and clinicians and quick to complete. However, there is a discrepancy in the comparison between patient self-reports and physician assessment of pain and physical function [8,41]. Furthermore, the questionnaires contain fixed response categories. This format can introduce problems of interpretation of the response scales in relation to the problem being evaluated. However, to choose the ideal health outcome measure is not an easy task [13]. They reflect different aspects of functionality and ability to develop activities, but not how patients perform these activities [12]. It is therefore necessary to use complementary measurements systems to assess biomechanical changes after the implantation of prosthetic components and throughout recovery, especially in weight loading asymmetry [7,16-21,42,43].

Patients with degenerative musculoskeletal disorders suffer from limitations in their walking ability [44]. Some studies indicate that gait mechanics do not return to normal after THA [7,45]. Gait velocity has been widely studied in patients after THA [12,46]. In our study, gait velocity was significantly higher after surgery compared with the pre-operative measurement session. GRF and time parameters showed significant improvement after THA. A significantly shorter stance time for the uninvolved lower limb was observed after surgery. This change of relative stance/swing phase duration suggests that before surgery, the patient uses his uninvolved leg as an extra support for the injured lower limb and this is corrected after operation. Higher average vertical GRF during walking with both lower limbs was observed after surgery than before THA (p ≤ 0.05). Given the change in gait velocity, a difference in vertical GRF could be expected in agreement with others [47-49]. In our study, a higher gait velocity indeed resulted in higher average GRF. This demonstrates that the gait pattern is more dynamic, active and vigorous after surgery than before*.* In a previous cross-sectional study with these patients [34], a correlation between ground reaction force and time parameters during walking and gait velocity was observed but there was no correlation between symmetry index parameters and gait velocity. Therefore, it was concluded that the asymmetry parameters provide information independent from gait velocity. Symmetry index of stance time and average vertical GRF during walking were negative before surgery, indicating that the non-involved leg was loaded for a longer period of time than the involved limb and that patients put more weight on the non-affected lower limb throughout the gait cycle. After surgery these symmetry indices were significantly different for both parameters (t_stance_ and A_vGRF,w_). The symmetry index of t_stance_ did not show asymmetry after surgery, however, the symmetry index of A_vGRF,w_, although smaller, still showed a significant asymmetry towards the uninvolved lower limb after surgery. Consequently, the change of symmetry index relative to the pre-surgical value provides important additional information about the recovery of these patients.

Many studies measured the capacity to perform the activity of walking [12,16,43]. However, patients also have problems with rising from a chair before and after THA [12]. In contrast with the significant recovery that gait performance indicates, the IS parameters measured during the STS test, maximum peak vertical GRF and dynamic area, did not indicate significant differences between both conditions, before and after THA. Patients put more weight on the non-affected leg throughout the STS movement before and after surgery. Moreover, the inter-limb asymmetry during STS test did not show significant improvement after surgery. Symmetry index of maximum peak vertical GRF and symmetry index of dynamic area were significantly asymmetric before and remained asymmetric after surgery. Apparently, THA does not result in a symmetric execution of this high demanding task, probably because it is a very difficult and challenging task with high loading [50]. As Talis has reported, the quantity of asymmetry in THA patients does not necessarily have to be the same during different tasks [7].

The meta-analysis of Vissers indicates that 8 months after surgery patients have already recovered about 80% of the preoperative levels. Nevertheless, it is not clear whether or when patients will recover to more than this level [12]. Our results on questionnaires, gait velocity and gait performance indicate that patients improved their functional capacity but the STS test showed no significant differences before and after surgery. This could imply that the recovery may not be as good as these other methods report. Our results are in agreement with those of Talis et al., when they compared THA patients with controls concluding that more demands are placed on the hip when rising from a chair than during walking [7]. This could indicate that during walking, patients may try to maintain their functional capacity as normal as possible despite the pain, muscle weakness and discomfort. However, while rising from a chair, the patients are used to unload their operated limb after the surgery and continued doing this during recovery. It is currently unclear whether these asymmetries will persist or disappear gradually during the post-operative time and whether they lead to overloading of the unaffected side, thereby promoting degeneration of the joints on this side.

Several published investigations agreed that 6 months post-surgery the recovery is demonstrated by an improvement in body mobility during walking but the sit to stand task has been reported not to improve to the same extend [7,51]. There are not many studies investigating the asymmetric lower limb loading to follow the evolution of these patients during rehabilitation. In future research, subsequent studies need to be performed to investigate the clinical relevance with a wider range of subjects after THA and during rehabilitation process*.* Moreover, it is important to design tailor-made rehabilitation programs and study whether the patients are able to execute more symmetric movement patterns after training, especially during highly demanding tasks like transferring from sit to stance. Patients are expected to benefit from the results of our study in future, because functional mobility performance can be assessed quantitatively with only one portable measurement system in a clinical setting. This can help clinicians and physiotherapist to optimize and evaluate the rehabilitation progress in the individual patient.

## Conclusions

We conclude that shoes with force and inertial movement sensing devices are sufficiently sensitive to demonstrate differences before and after THA. Inter-limb asymmetry can be evaluated with the IS supplying important information which is clinically relevant in the screening before and during rehabilitation after THA. This makes it a new clinical measurement concept useful for tracking the evolution of hip OA patients before and after THA in a regular clinical setting.

## Abbreviations

THA: Total hip arthroplasty; OA: Osteoathritis; HHS: Harris hip score; WOMAC: Traditional Western Ontario and McMaster Universities osteoarthritis index; STS: Sit to stand; IS: Instrumented shoes; METC: Medical Ethics Committee; GV: Gait velocity; BW: Body weight; tstance: Stance time; GRF: Ground reaction force; AvGRF,w: Average vertical ground reaction force; trise: Rise time; PvGRF,sts: Maximum peak vGRF; DvGRF,rise: Dynamic area; SI: Symmetry index; SD: Standard deviation; CI: Confidence intervals.

## Competing interests

The authors declare that they have no competing interests.

## Authors’ contributions

AM-R: Conception and design, acquisition, analysis and interpretation of data; drafted the article. DW: Acquisition of data. PL: Drafted the article. NV: conception and design. DP: conception and design, acquisition of data. PHV: conception and design, drafted the article. All the authors revised critically the article for important intellectual content and gave the final approval of the version to be published. All authors read and approved the final manuscript.
